# Poisons

**Published:** 1855-03

**Authors:** 


### POISONS.

We all have a great horror of being poisoned, without exactly understanding what it is.

*Poison is a disorganization of flesh, or blood, or both.*

Poisons are of two kinds. One, the result of medicinal agents taken into the stomach or circulation, the other the result of bites or stings of living creatures.

I will now state two ideas, which if generally known, and remembered, would save thousands of lives every year.

If you have swallowed a poison, whether laudanum, arsenic, or other thing poisonous, put a table-spoon of ground mustard in a glass of water, cold or warm, stir and swallow quickly, and instantaneously the contents of the stomach will be thrown up, not allowing the poisonous substance time to be absorbed and taken into the blood, and as soon as vomiting ceases, swallow the white of one or two new eggs, for the purpose of antagonizing any small portion of the poison which may have been left behind. Let the reader remember the principle, which is to get the poison out of you as soon as possible; there are other things which will produce a speedy emetic effect, but the advantage of mustard is, it is always at hand, it acts instantaneously, without any after medicinal effects.

The use of the white of an egg is, that although it does not nullify all poisons, it antagonizes a larger number than any other agent so readily attainable.

But while taking the mustard, or egg, send for a physician; these are advised in order to save time, as the difference of twenty minutes is often death.

CURE OF BITES AND STINGS.

Almost all these are destructive from their acid nature: consequently the cure is an alkali. Spirits of Hartshorn is one of the strongest, and in almost every house, and you have only to pour out some into a tea-cup, and dabble it on the wound with a common rag; relief is almost instantaneous. But suppose you have no Hartshorn, well, then, Saleratus is an alkali, every trifling lazy cook in the land has it, we are daily eating ourselves into the grave by its extravagant use, and the use of half a thimble-full in a week is extravagant. Moisten it with water, and use as the Hartshorn. If you have no Saleratus or Soda, pour a teacup of boiling water on as much wood ashes, stir it, and in a few moments you will have an alkali. The ley of ashes will answer a good purpose while the physician is coming. Remember the principle, the bite is an acid, the cure is an alkali.

Have we not before now looked with wonder on the old negro, who ran out when the wasp's sting made us "holler," caught up "*three kinds*" of weed, rubbed the part well, and in five minutes we were happy in the complete relief. But why "*three*" kinds of weed? Why, in the first place, you know "three" and all its multiples are mysterious numbers; and then again, you can scarcely gather up three kinds of plants anywhere, one of which will not have more or less of alkali in it. If men were only to gather up *principles* instead of *specifications*, how much easier it would be to know a great deal, and to apply our knowledge successfully to the practical purposes of life.

